# Japanese primary care physicians’ experience in treating their family members: a cross-sectional study

**DOI:** 10.1186/s12875-022-01848-y

**Published:** 2022-09-16

**Authors:** Taku Matsunaga, Makoto Kaneko, Michael D. Fetters, Machiko Inoue

**Affiliations:** 1 Shizuoka Family Medicine Program, Hamamatsu University School of Medicine, Shizuoka, Japan; 2 Morimachi Family Medicine Clinic, 387–1, Kusagaya, Morimachi, Shuchi-Gun, Shizuoka, 437–0214 Japan; 3 Department of Health Data Science, Yokohama City University, Kanagawa, Japan; 4 Mixed Methods Program and Department of Family Medicine, University of Michigan, Ann Arbor, Michigan, USA; 5 Department of Family and Community Medicine, Hamamatsu University School of Medicine, Shizuoka, Japan

**Keywords:** Treating family members, Physicians, Primary care, Family practice, General practice

## Abstract

**Background:**

Guidelines worldwide recommend that physicians should not treat their family members. However, studies in the U.S. have shown that approximately 74–83% of physicians have experience of treating family members. Primary care physicians were more likely to have such experiences than other specialists. In Japan, physicians do not have any guidelines regarding treating family members, and little is known about the experiences of primary care physicians. Therefore, we investigated the experience of treating family members or relatives among primary care physicians in Japan.

**Methods:**

This cross-sectional study used an online questionnaire. We recruited 2,000 physicians who were members of the Japan Primary Care Association using random sampling. Data were collected from February 10 to March 10, 2021. We compare the experiences of treating family members between clinic-based doctors and hospital-based doctors using the chi-square test. We performed logistic regression analysis to adjust for gender, age, presence of a doctor in family, and physician’s geographic location (rural or not rural).

**Results:**

A total of 466 physicians (response rate = 23.3%) completed the survey. Of the sample, 79.8% had experience of treating family members or relatives. In the univariate analysis, being a clinic-based physician was associated with experience in treating family members compared to hospital-based physicians (87.6% vs. 74.9%, *p* = 0.001). Multivariable analysis showed that being a clinic-based physician (odds ratio 2.30, 95% confidence interval 1.31–4.04) and age of 45–64 years (odds ratio 2.93, 95% confidence interval 1.74–4.93) were significantly related to experience treating family. Gender and geographic location were not statistically significant factors.

**Conclusions:**

A high percentage of Japanese primary care physicians, especially those who worked in clinics, reported experience treating family members or relatives. These findings will serve as basic data for future studies regarding the care of families and relatives of physicians in Japan.

## Background

For physicians, providing medical care to family members and relatives raises ethical and clinical issues [1, 2]. The reasons are a possible lack of objectivity, potential failure to ask about sensitive topics or to perform intimate examinations, risk of practicing outside the scope of training, over testing, and inappropriate prescribing [1–4]. The American Medical Association Code of Medical Ethics guidelines state that “In general, physicians should not treat themselves or members of their own families [3].” Similarly, the American College of Physicians recommends not entering into dual relationships, referring to patient-physician relationships and family-physician relationships [4]. The Canadian Medical Association asks physicians to limit the treatment of their immediate family to minor or emergency interventions and only when another physician is not readily available [5]. The UK’s General Medical Council also asserts that physicians must avoid prescribing to anyone they have a close personal relationship with whenever possible [6].

However, a study by La Puma showed that more than 99% of physicians had received requests from family members asking for medical advice, diagnosis, or treatment, and 83% of physicians had prescribed for relatives [7]. This study revealed that primary care physicians provided more services to families or relatives than physicians in other specialties. Previous studies in the US also showed that 74–80% of physicians had treated their family members [8–10]. In a German study, 96.7% of the GPs had treated at least one family member in the last 12 months [11].

In Japan, physicians do not have guidelines regarding treating their families and relatives, and little is known about the experiences of primary care physicians. As in other countries, the number of physicians with experience is not expected to be small, but there has been no previous research on the actual situation.

Therefore, the purpose of this study was to investigate the experience of treating family members or relatives among primary care physicians in Japan.

## Methods

### Study design

We performed a cross-sectional study using an online questionnaire.

### Setting and participants

Participants were physicians who were members of the Japan Primary Care Association (JPCA). The JPCA is an academic organization which comprises many Japanese primary care physicians and has around 10,470 members (as of February 2019). We recruited 2,000 physicians using random sampling, who were at least three years postgraduate. The first and second years after graduation from medical school fall under the category of initial postgraduate clinical training [12], and were thus excluded.

In Japan, primary care services are provided by both clinics and hospitals [13]. Also, the boundary between primary care and specialty areas is ambiguous and the census reports that approximately 32% of physicians work in clinics and 64% work in hospitals among all physicians [14]. Therefore, the respondents included physicians working in hospitals.

In addition, there are two important characteristics of the healthcare system in Japan, which are universal health insurance and free access [13]. All residents of Japan are required by law to be enrolled in a health insurance program, and co‐payment rates range from 10 to 30%. Patients are free to choose any healthcare facility, regardless of the severity of their disease and their insurance status.

### Study period

Data were collected from February 10 to March 10 in 2021.

### Data collection

Participants answered the questionnaire on the Web using Microsoft Forms. We provided URLs via email and sent a total of three reminder emails over the period of data collection. The first 300 respondents received an Amazon gift certificate worth JPY 2,000 (approximately USD 17.4).

### Primary outcome: having experience in treating their family members or relatives

The following question was used to explore participants’ experience in treating family members. “Looking back over the past few years, have you ever provided medical care (including medical examinations, prescriptions, procedures, etc.) to your family members or relatives at least once?”.

Based on past studies and reports, we defined “family members or relatives” to include parents, children, siblings, grandparents, grandchildren, spouses, and spouses’ first-and second-degree relatives [2, 7, 15].

### Explanatory variable: field of practice (clinic or hospital)

Participants chose their fields of practice from the following: clinics without admission, clinics with admission (1–19 beds), small hospitals (less than 200 beds), medium hospitals (200–499 beds), large hospitals (500 beds or more), and others. In Japan, a hospital is defined as a medical institution with 20 beds or more, whereas a clinic is defined as having less than 20 beds or no beds. In addition, there is a difference in the medical fees among hospitals with more than 200 beds and those with less than 200 beds. Therefore, this classification was used in a previous study conducted in Japan [12].

### Covariates

Based on the previous studies, we included covariates for age, gender, geographic location, and whether or not physicians had relatives who were doctors [7, 10, 15]. These covariates were evaluated as categorical variables through a self-administered questionnaire.

### Types of treatment, reasons and factors behind the treatment, and physicians’ feelings during the treatment

We also collected information on the type of medical treatment families and relatives received, the reasons and factors behind the treatment, and their feelings (satisfaction or hesitation) during treatment. Some of the questions were: “What were your reasons for providing medical care to your family or relatives?;” “Have you felt satisfied/hesitant while treating family or relatives?” For the latter question, we asked participants to choose from “Often,” “Sometimes,” “Rarely,” and “Never.” These questions and alternatives were based on past studies [7, 15].

### Statistical analysis

Nominal data were expressed as percentages, whereas medians with interquartile ranges were calculated for continuous variables. Responses were excluded if there were missing or apparent inconsistencies in them.

We conducted a chi-square test with field of practice (binary variable of hospital or clinic) as the independent variable, and the percentage of doctors who had treated family members or relatives as the outcome. In addition, we conducted a logistic regression analysis with the following factors: age, gender, physician’s geographic location, and presence of a doctor in the family. Age was classified into three groups: < 45 [7], 45–64, ≥ 65 years, and included in the model as a categorical variable.

As four variables, that is, age, gender, physician’s geographic location, and presence of a doctor in the family were included in the final model, and the percentage of “having experience in treating family members or relatives” was estimated to be 80% from previous studies, we calculated that we would require at least 250 responses for this study. All statistical analyses were conducted using StataCorp software (Stata Statistical Software, Release 15, College Station, TX, StataCorp LLC, 2017).

## Results

In total, 468 physicians responded to the survey. Two respondents with inappropriate answers regarding age were excluded from the study, so the data of 466 was used (response rate = 23.3%). Table 1 presents the demographic characteristics of the participants. Women accounted for 20.8% of the total participants, men for 78.1%. The age range was 27–80 years with a median age of 44 years (interquartile range: 37–54 years). Of the respondents, 161 (34.5%) worked in clinics, 299 (64.2%) in hospitals, and 6 (1.3%) in others. In terms of geographic location, 59 (12.7%) were in remote islands or rural areas, and 407 (87.3%) were in neither of these locations.

**Table 1:** Demographics of the participants (*n* = 466)

	With experience in treating family members or relatives *n* = 370: n (%)	Without experience in treating family members or relatives *n* = 96: n (%)
Gender		
Man	295 (79.7)	69 (71.9)
Woman	71 (19.2)	26 (27.1)
Others	1 (0.3)	1 (1.0)
No response	3 (0.8)	0
Age (year)		
< 45	171 (46.2)	70 (72.9)
45–64	180 (48.6)	24 (25.0)
≥ 65	19 (5.1)	2 (2.1)
Field of Practice		
Clinic without admission	130 (35.1)	18 (18.8)
Clinic with admission (1–19 beds)	11 (3.0)	2 (2.1)
Hospital (20–199 beds)	99 (26.8)	19 (19.8)
Hospital (200–499 beds)	59 (15.9)	27 (28.1)
Hospital (500 beds or more)	66 (17.8)	29 (30.2)
Others	5 (1.4)	1 (1.0)
Geographic location		
Isolated island	7 (1.9)	3 (3.1)
Rural area	40 (10.8)	8 (9.4)
Not isolated island nor rural area	323 (87.3)	84 (87.5)
Presence of a doctor in family		
Yes	162 (43.8)	30 (31.3)
No	208 (56.2)	66 (68.8)

The total number of physicians with experience in treating family members and relatives was 370 (79.4%). The percentage of physicians treating family members or relatives was significantly higher in the clinic group than in the hospital group (87.6% vs. 74.9%, *p* = 0.0014).

Table 2 shows the results of the multivariable analysis. Working in a clinic (OR 2.30, 95% CI 1.31–4.04) and the age group of 45–64 years(OR 2.93, 95% CI 1.74–4.93) were significantly associated with experience. There were no associations with gender, presence of a doctor in the family, or geographic location (rural or not rural).

**Table 2:** Results of multivariable analysis of treating family members or relatives

	Crude odds ratio	*P*-value	95% CI^b^	Adjusted^a^ odds ratio	*P*-value	95%CI^b^
Clinic-based	2.34	0.002	1.37–4.0	2.30	0.004	1.31–4.04
Woman	0.64	0.091	0.38–1.07	0.74	0.27	0.43–1.27
Age						
< 45 (reference)						
45–64	3.07	0.000	1.85–5.11	2.93	0.000	1.74–4.93
≥ 65	3.89	0.073	0.88–17.14	3.88	0.077	0.86–17.48
Rural	1.02	0.96	0.52–2.01	1.09	0.82	0.53–2.22
Presence of a doctor in physician’s family	0.58	0.027	0.36–0.94	1.54	0.09	0.93–2.53

^a^ We adjusted for gender, age, physician’s geographic location (rural or not rural), and presence of a doctor in their family

^b^ *CI* Confidence Interval

**Fig. 1:**
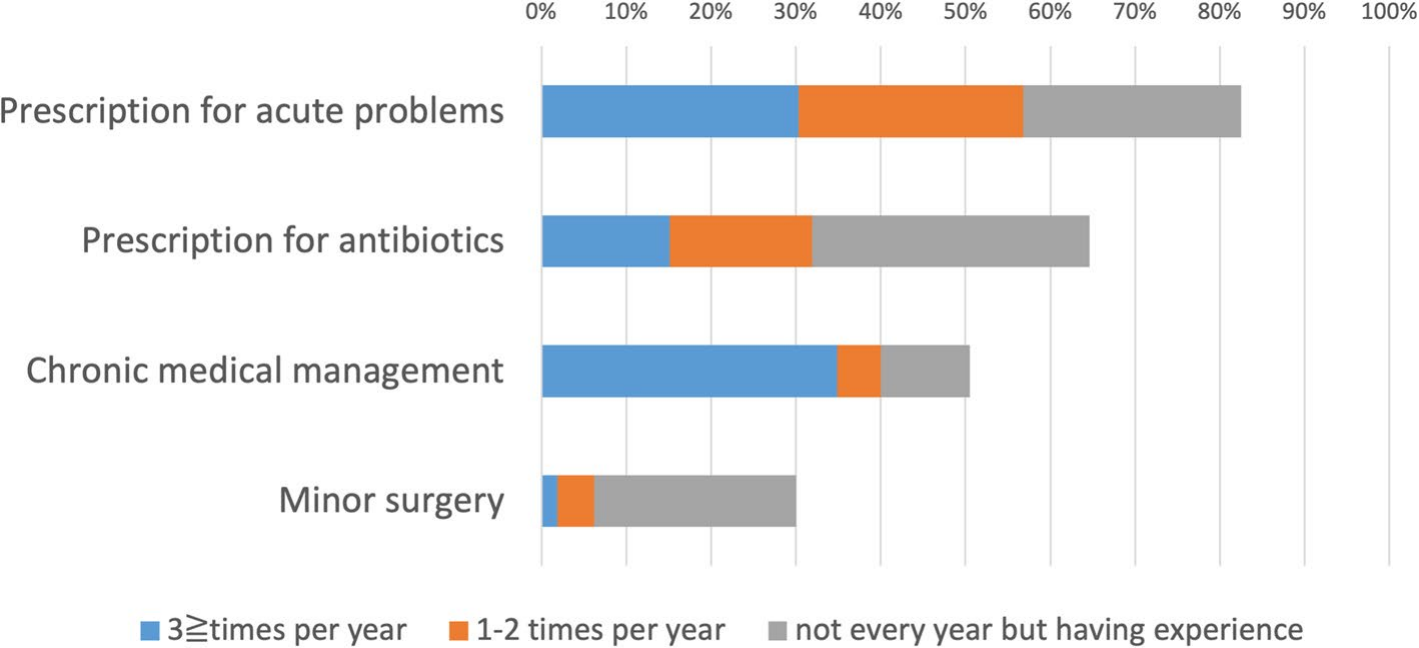
Types of experience in treating family members or relatives (1) (*n* = 370). Legend: The percentages of those with experience in prescribing for acute problems, antibiotic prescribing, chronic medical management, and minor surgery were 82.4%, 64.6%, 50.5%, and 30.0%, respectively

**Fig. 2:**
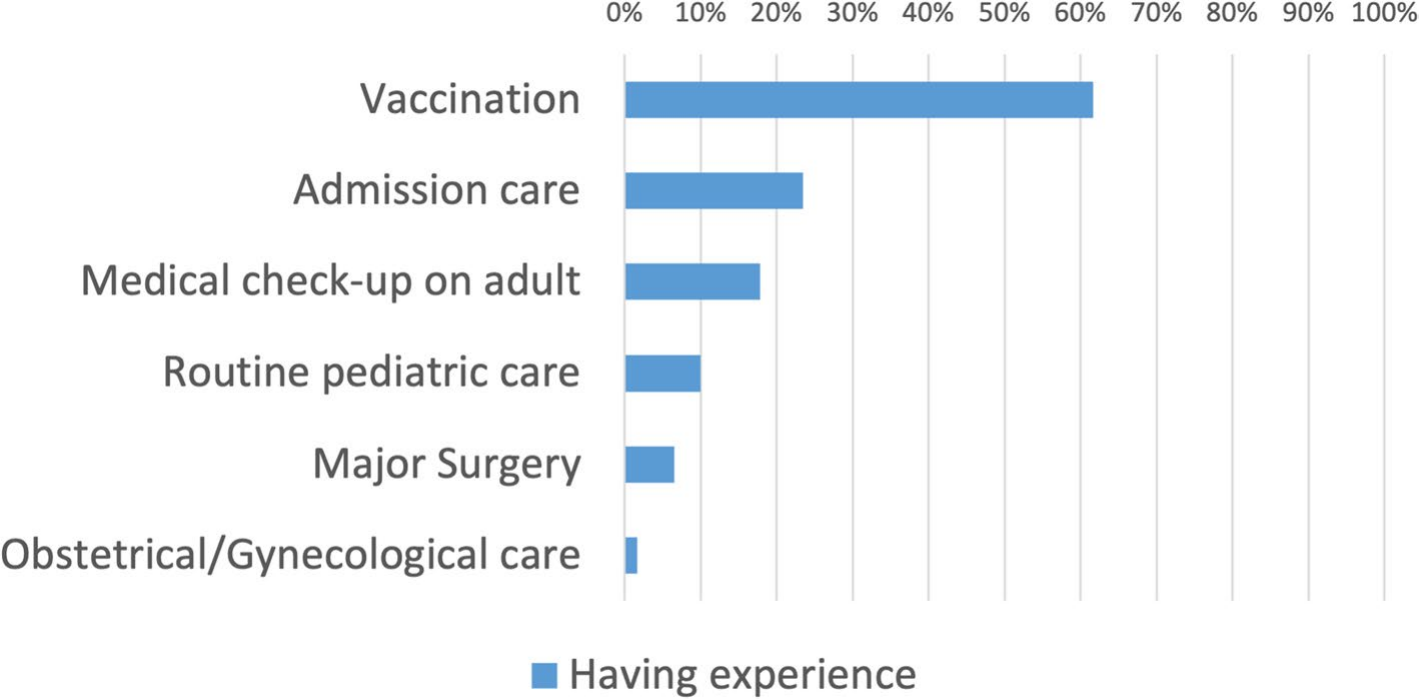
Types of experience in treating family members or relatives (2) (*n* = 370). Legend: The percentages of those who had experience with vaccine injections, admission care, medical check-up on adult, routine pediatric care, major surgery, and obstetric/gynecological care were 61.6%, 23.5%, 17.8%, 10.0%, 6.5%, and 1.6%, respectively

Figures 1 and 2 illustrate the types of medical care provided. Prescription rates for acute and chronic diseases were 82.4% and 50.5%, respectively. Vaccination was 61.6%, routine pediatric care was 10.0%, and medical checkups for adults was 17.8%. Obstetric or gynecological examination, including pelvic examination, was performed by 1.6% of the participants.

**Fig. 3:**
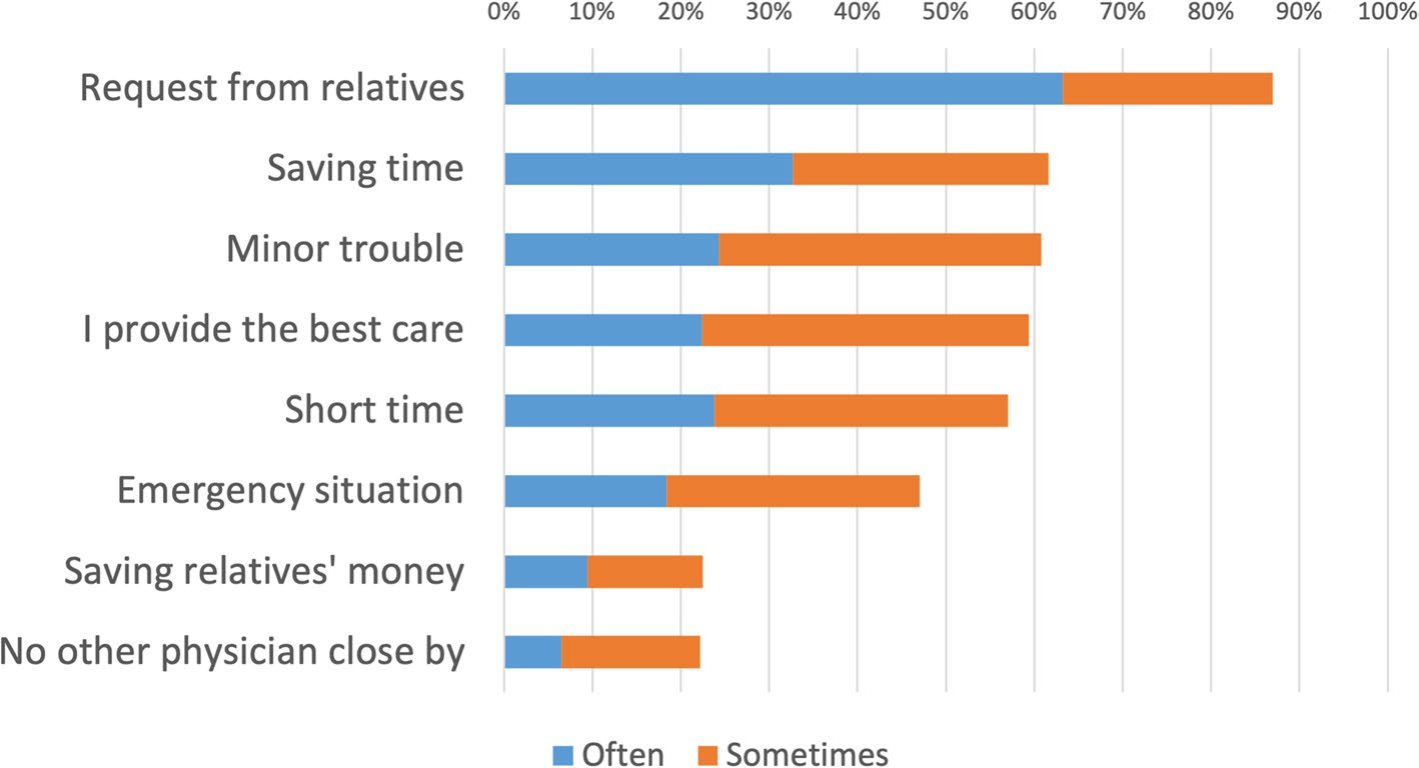
Reasons for treating family members or relatives (*n* = 370). Legend: The question was “What were your reasons for providing medical care to your family or relatives.” The respondents were asked to choose “Often”, “Sometimes”, “Rarely”, or “Never” for each of them. This figure shows the results for “Often” and “Sometimes” combined

**Fig. 4:**
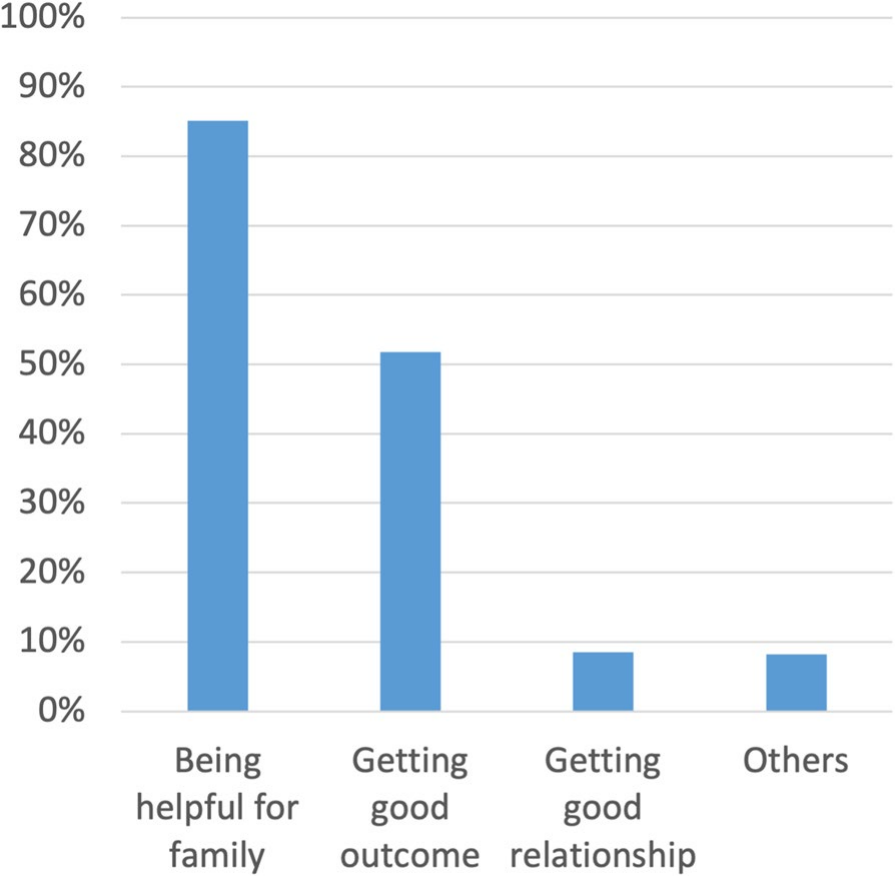
Reasons for satisfaction in treating family members or relatives (*n* = 330). Legend: Results of a multiple-answer question regarding the reasons for satisfaction in treating family members or relatives

**Fig. 5:**
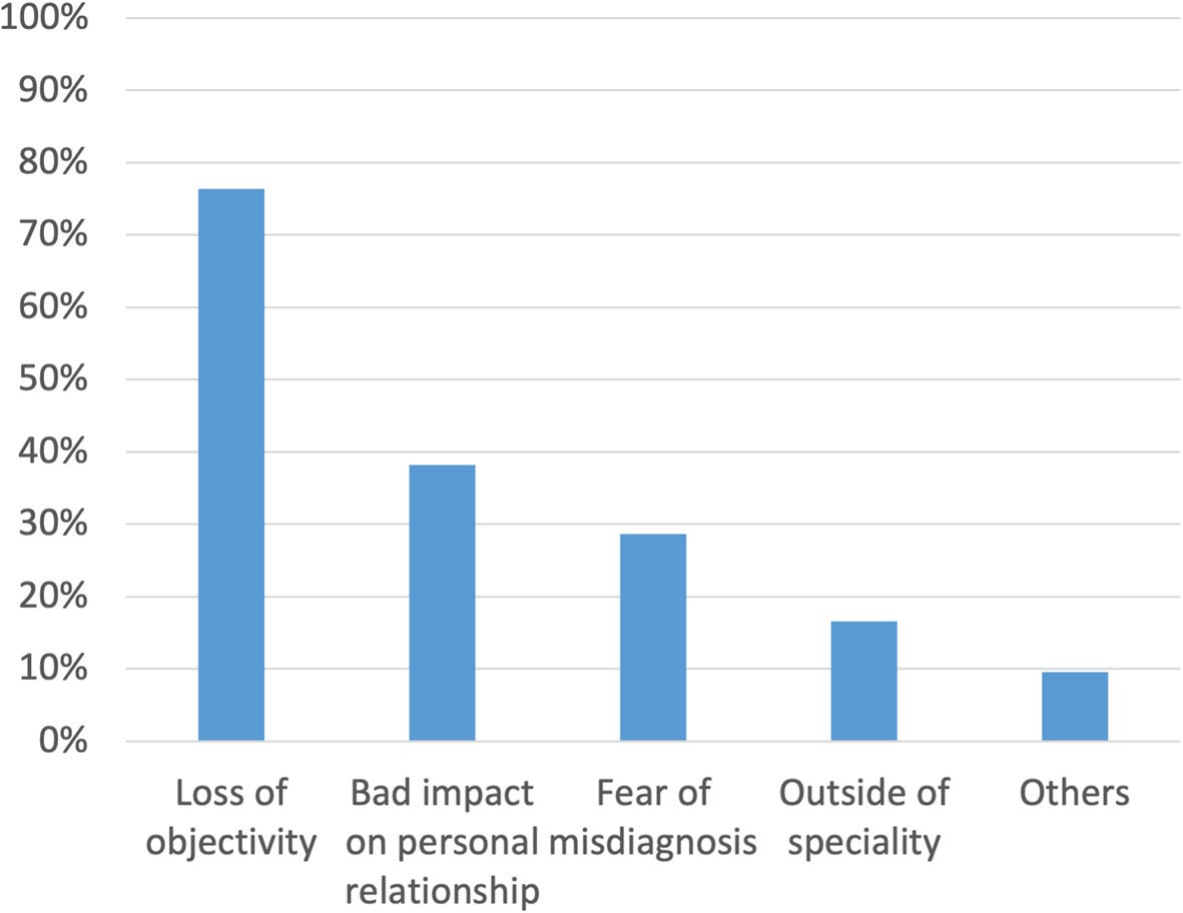
Reasons for hesitation in treating family members or relatives (*n* = 157). Legend: Results of a multiple-answer question regarding the reasons for hesitation in treating family members or relatives

Figure 3 shows the reasons for providing medical care to families. The physicians who were satisfied with the treatment comprised 89.2%, including the “Often” and “Sometimes” responses. The percentage of physicians who were hesitant during treatment was 42.4%. The reasons for satisfaction and hesitance with providing medical care are shown in Figs. 4 and 5, respectively.

In addition, 72.5% of the respondents did not know about the guidelines in Europe and the US for the treatment of family members and relatives, and 45.1% of the respondents agreed or somewhat agreed, 20.4% disagreed or somewhat disagreed, and 34.5% were undecided regarding the creation of guidelines in Japan.

## Discussion

In this study, we found that about 80% of primary care physicians in Japan had experience of treating family members, which is similar to the findings of previous studies in the US. We had considered the possibility that the percentage of physicians treating family members might be higher than in other countries due to Japan’s universal health insurance and free access, but the results showed almost the same numbers.

The proportion of physicians with experience in treating family members or relatives was significantly higher in the clinic group than in the hospital group. In previous studies, convenience, saving costs, and having greater knowledge and concern than their colleagues were cited as the reasons for providing medical care to family members [10]. In this study, the most common reason for providing care was “request from family or relatives.” We assume that higher accessibility compared to hospitals results in a higher percentage of medical experience in family treatment in the clinic group.

In previous studies, age ≥ 45 years was associated with more experience in providing medical care to family members [7], and the logistic regression analysis in this study also showed that a relatively higher age was a significant factor. Considering that the most common reason for providing medical care is a request from family members or relatives, it is natural that an increase in the number of opportunities to be asked will increase experience. Moreover, for physicians aged ≥ 65 years, such opportunities may decrease. The types of medical care for family members or relatives were similar to those reported in previous studies [7, 15].

Regarding the reasons for providing medical care to families, the Malaysian qualitative study cites the influence of the socio-cultural milieu in Malaysia, where families are expected to help each other [16]. Given that the most common reason for treating family members or relatives was the request from them in this study, such influence may apply to Japan as well. In the study by Reagan, saving relative’s money was the third of the options as a reason for treating family members, but in this study it was the second from the bottom of the options [15]. This may be due to Japan’s universal health insurance, which may have influenced the results.

We did not expect that the percentage of physicians who felt satisfied with the care of family members would reach nearly 90%, whereas the percentage of physicians who felt hesitant was merely 40%. Regarding the development of the guideline, 45.1% of the respondents agreed or somewhat agreed, 20.4% disagreed or somewhat disagreed, and 34.5% were undecided. These results might explain the reason why Japanese primary care physicians have never created the guideline before.

### Study strengths

This is the first study to identify the experiences of Japanese physicians in treating their family members or relatives. The number of respondents was relatively large compared to previous studies. Furthermore, to the best of our knowledge, no previous study has evaluated the differences in experiences between those working in clinics and those working in hospitals. The results of the study might be useful for other countries where primary care physicians involve inpatient care such as the US and Canada [17, 18].

### Study limitations

Our study has several limitations. First, physicians who had a greater interest in treating their family members might have responded to the survey in greater numbers, leading to an overestimation of the actual rate. However, the rate of about 80% is similar to that observed in previous studies, and we consider the result to reflect reality in Japan. Second, the study was limited to physicians who were members of the JPCA. The number of members is approximately 10,000 among a total of more than 300,000 physicians in Japan. As the definition of primary care is ambiguous in Japan [13], we chose the most representative academic organizations in Japan to minimize this bias. However, the results need to be carefully extrapolated to other primary care societies such as the Japan Society of Hospital General Medicine or other specialties. Third, we were unable to track the outcomes of patients after treatment. This issue needs to be addressed in the future because no previous research has quantitatively tracked patient outcomes, although several qualitative studies have revealed good and bad outcomes or changes in relationships [16, 19–21].

## Conclusions

As in other countries, nearly 80% of primary care physicians in Japan have treated family members or relatives, especially those who are clinic-based. These findings should stimulate discussions regarding family treatment by primary care physicians and serve as basic data for future studies on the care of physicians’ families or relatives.

## Data Availability

The datasets generated and analysed during the current study are not publicly available because we did not receive informed consent concerning data sharing from the participants, but the datasets are available from the corresponding author on reasonable request.

## Abbreviation

JPCA: Japan Primary Care Association
